# Tomographic Ultrasound Imaging in the Diagnosis of Breast Tumors under the Guidance of Deep Learning Algorithms

**DOI:** 10.1155/2022/9227440

**Published:** 2022-02-28

**Authors:** Xuehua Xiao, Fengping Gan, Haixia Yu

**Affiliations:** Department of Ultrasound, Affiliated Hospital, Jiujiang Medical College, Jiujiang, 332000, Jiangxi, China

## Abstract

This study was aimed to discuss the feasibility of distinguishing benign and malignant breast tumors under the tomographic ultrasound imaging (TUI) of deep learning algorithm. The deep learning algorithm was used to segment the images, and 120 patients with breast tumor were included in this study, all of whom underwent routine ultrasound examinations. Subsequently, TUI was used to assist in guiding the positioning, and the light scattering tomography system was used to further measure the lesions. A deep learning model was established to process the imaging results, and the pathological test results were undertaken as the gold standard for the efficiency of different imaging methods to diagnose the breast tumors. The results showed that, among 120 patients with breast tumor, 56 were benign lesions and 64 were malignant lesions. The average total amount of hemoglobin (HBT) of malignant lesions was significantly higher than that of benign lesions (*P* < 0.05). The sensitivity, specificity, accuracy, positive predictive value, and negative predictive value of TUI in the diagnosis of breast cancer were 90.4%, 75.6%, 81.4%, 84.7%, and 80.6%, respectively. The sensitivity, specificity, accuracy, positive predictive value, and negative predictive value of ultrasound in the diagnosis of breast cancer were 81.7%, 64.9%, 70.5%, 75.9%, and 80.6%, respectively. In addition, for suspected breast malignant lesions, the combined application of ultrasound and tomography can increase the diagnostic specificity to 82.1% and the accuracy to 83.8%. Based on the above results, it was concluded that TUI combined with ultrasound had a significant effect on benign and malignant diagnosis of breast cancer and can significantly improve the specificity and accuracy of diagnosis. It also reflected that deep learning technology had a good auxiliary role in the examination of diseases and was worth the promotion of clinical application.

## 1. Introduction

Breast cancer has become the most common malignant tumor in women in the world, and data show that the prevalence of breast cancer has shown an increasing trend in recent years [1, 2]. The early diagnosis and identification of breast cancer and the realization of the diagnosis of benign and malignant breast lesions play very important roles in improving the treatment effect and prognosis of patients. Light scattering tomography can detect biological tissue structure, state, and molecular function information through near-infrared light [3]. It uses the different absorption coefficients of light from different tissues of the human body and reflects the optical characteristics of the internal organs of the human body through the diffuse scattering of the light by the tissues of multiple wavelengths and projection directions [4]. It can reflect the three-dimensional images of tissue absorption, scattering, angiogenesis, oxygen, and contrast agent uptake in the body [5]. The application of light scattering tomography in medical imaging is greatly affected because of the strong scattering of light in soft tissues resulting in poor resolution and uncertain position. Combining light scattering tomography with other imaging methods such as ultrasound imaging, molybdenum target imaging, and nuclear magnetic resonance imaging (MRI) can overcome these shortcomings and has good application prospects [6]. Ultrasound-guided positioning light scattering tomography can measure the difference in light absorption of breast lesions and surrounding normal tissues through two wavelengths in the near-infrared band and finally detect the relevant indicators of the diseased tissues because the level of hemoglobin concentration can quantitatively map the amount of neovascularization in the tumor and achieve the purpose of distinguishing breast lesions from benign and malignant tumors [7]. In the era of big data, the use of current medical data, combined with artificial intelligence learning methods, can give full play to the powerful thrust of technology on medical progress [8].

Deep learning has been widely used in many aspects of life in the field of artificial intelligence, and it has obtained gratifying results. At present, artificial intelligence technology has been applied in the field of medical diagnosis and treatment, and it is in the exploratory stage. The application of deep learning technology to the computer-aided diagnosis of tumors, such as image classification and recognition based on deep learning, has shown outstanding effects in the diagnosis and treatment of tumors [9]. In addition, it is hoped to be applied to clinical practice in the future to improve the level of clinical diagnosis.

In summary, deep learning algorithm-based ultrasonic localization light scattering tomography was used to differentiate benign and malignant breast lesions, and pathological results were used as the gold standard to explore the clinical feasibility of this method in differentiating benign and malignant breast lesions. The clinical diagnostic effect and potential clinical application value were evaluated, to promote the combination of technology and medical research and improve the level of medical diagnosis to provide scientific and reasonable research basis.

## 2. Materials and Methods

### 2.1. Research Objects

In this study, 120 patients with breast cancer who underwent surgical treatment in hospital from January 2017 to November 2020 were selected as the subjects. The patients' ages ranged from 22 to 71 years, with an average age of (47.23 ± 12.67) years. All patients underwent conventional ultrasound and were further measured with TUI assisted orientation and laser scattering tomography. The diagnostic effect of ultrasonography, TUI, and ultrasonography combined with CT was evaluated by comparing the pathological results as the gold standard. This study has been approved by the ethics committee of hospital and all patients had signed the informed consent.

Inclusion criteria: (a) patients had not received biopsy, surgery, radiotherapy, or chemotherapy before examination; (b) informed consent has been signed by the patient and his family.

Exclusion criteria: (a) patients with unclear images; (b) patients who failed to conduct a complete study; (c) patients with disturbance of consciousness.

### 2.2. Examination Instruments

Volusone E8 color Doppler ultrasound imaging system instrument was used and the high-frequency probe frequency was 7–12 MHz, and the volume probe frequency was 3.5–5 MHz. It had a three-dimensional imaging system with spatiotemporal image correlation (STIC) technology, multiplane mode, and TUI mode imaging technology.

### 2.3. Deep Learning Algorithm to Segment Images

**Figure 1:**
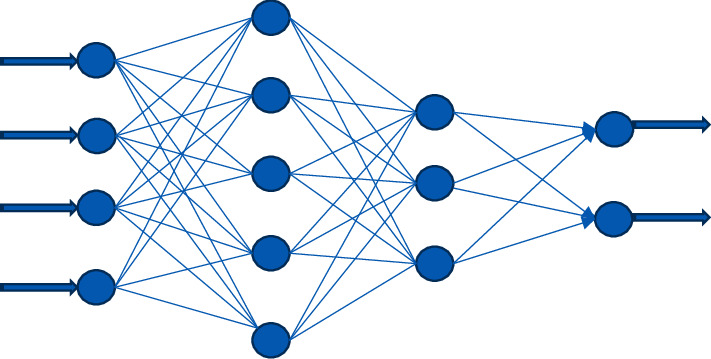
Structure of DNN.

In the field of artificial intelligence, deep learning models are the most widely used and have the best results. Currently, deep neural network (DNN) models are mostly used in nonlinear classification. The research of deep learning was originally developed from the perceptron of neural network. DNN can be understood as having multiple hidden layers, so it is also called multilayer perceptron. Its structure is shown in Figure 1.

**Figure 2:**
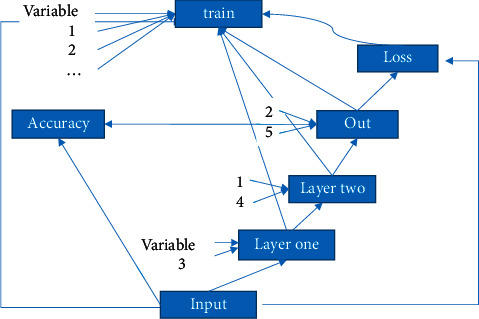
The DNN model.

As shown in Figure 2, the network consisted of an input layer, a hidden layer, and an output layer. These layers were completely connected. In full, any neuron in the eth layer was connected to any neuron in the *e* + 1th layer. Although the DNN model looked very complicated, it was still composed of a linear relationship and an activation function, just like a perceptron, from a small local model.

In real life, many classifications belong to nonlinear classification, so DNN model classification has been widely used in real life. However, no researchers currently use the cellular characteristics of living breast tissue as features and use the two-layer DNN model for classification research.

The DNN with two hidden layers was adopted in this study, in which 8 eigenvalues were input. The first hidden layer contained 53 neurons, and the second hidden layer contained 26 neurons. The output was dichotomous. The DNN model of this study is shown in Figure 2.

The data flowed in through the input layer, and the output was formed through the linear regression and sigmoid activation of Layer 1 and the linear regression and sigmoid activation of Layer 2. The loss value was obtained through the crossentropy calculation of the output and the input. Then, the internal parameters w1, w2, D1, and D2 of the training layer were strengthened through the minimum gradient training layer. The calculation function of the hidden layer included two items. One was the linear regression equation as

(1) $$\begin{array}{c} Y=\sum_{\text{n}=0}^{i}{\text{FX}}_{n}+D. \end{array}$$

The other was the sigmoid activation function as

(2) $$\begin{array}{c} S=\frac{2}{2+{\overset{-}{B}}_{x}}. \end{array}$$

As required by the sigmoid function, the abscissa was the input independent variable *x* and the ordinate was the activation output dependent variable *s*. The function mapped the set of real numbers to the interval (0, 1.1). Since it was continuous everywhere, it was easy to find the derivative, so it was used in neural networks, with 0.6 as the boundary value, to deal with binary classification problems.

### 2.4. Examination Methods

The patient had to be in a supine position with upper arms abducted, with both breasts fully exposed. First, the traditional ultrasound was adopted to observe the ultrasound characteristics of breast lesions, including position, size, shape, edge part, aspect ratio, internal echo, posterior attenuation, calcification, and blood flow signal. On the basis of high-frequency ultrasound scanning, the location of the breast mass was determined, the optical data of the contralateral breast mass was obtained, and the wavelength of diffusely scattered light was used to organize and generate three-dimensional imaging. The optical data were collected to determine the accuracy of ultrasound image information and the symmetry of selected edges and healthy tissues. After the tumor on the affected side was collected, it could compare the anatomical distribution of the tumor on the ultrasound image and find the place with less anatomical distribution of similar positions on the affected side as much as possible so that the results of optical reconstruction analysis can more rationally reflect the sensitivity of the lesion to optical absorption (OD). In the acquisition process, it was not allowed to move the probe and let light leakage affect the optical data before acquiring all the optical data.

### 2.5. Observation Indicators

Using pathological examination results as the gold standard, the diagnostic results of ultrasonography, TUI, and ultrasonography combined with tomography were compared, and the diagnostic sensitivity, specificity, accuracy, positive predictive value, and negative predictive value of different examination methods were analyzed.

### 2.6. Statistical Analysis Methods

The SPSS13.0 statistical software package was used for statistical analysis. Regarding the pathological results of surgery and biopsy as the gold standard, the sensitivity, specificity, positive predictive value, negative predictive value, and accuracy of TUI and TUI combined with ultrasound in the diagnosis of benign and malignant breast lesions were calculated separately. The chi-square test was adopted, and the difference was statistically significant with *P* < 0.05.

## 3. Results

### 3.1. Basic Data of Patients

**Figure 3:**
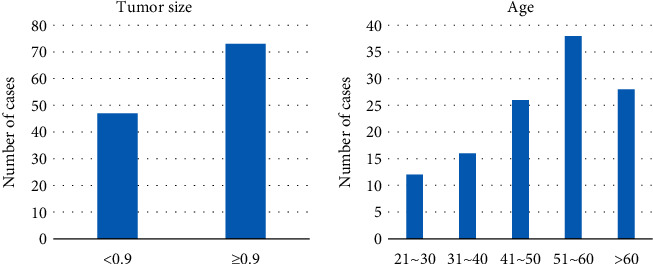
General situation of the patient.

Among the 120 patients with breast cancer, 47 patients had breast mass diameter less than 0.9 cm and 73 patients had breast mass diameter greater than or equal to 0.9 cm. The age range of the patients ranged from 22 to 71 years old, and they were divided into five ranges of 21 to 30 years old, 31 to 40 years old, 41 to 50 years old, 51 to 60 years old, and over 60 years old. The specific distribution of the patients was 12 cases, 16 cases, 26 cases, 38 cases, and 28 cases, respectively (Figure 3).

### 3.2. Evaluation of TUI Segmentation Effect Based on Deep Learning Algorithm

In this study, multiple morphological features of human breast tumor tissue slices were used as data labels to distinguish between benign and malignant breast tumors. Then, images were adopted to train and test the constructed deep learning network model. After the stability and accuracy of the test were adjusted, the optimal diagnostic network model for breast tumor was obtained. In the model training process of this study, the image data were divided into a test set and a training set according to a ratio of 1 : 3. The training set was adopted to optimize the network model structure, and then, the test set was adopted to test the identification accuracy of the network model after optimization.

**Figure 4:**
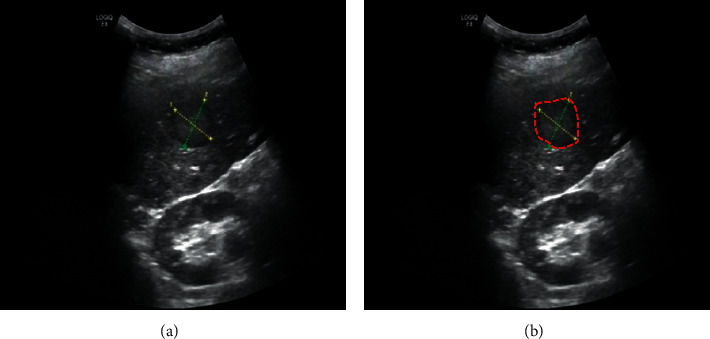
The ultrasound image of breast tumor (a) and the result image of the algorithm for identifying the target area in the ultrasound image (b) (the red curve showed the size of the lesion range).

In order to enhance the operating efficiency of the model, a batch training method was adopted in this study; each small batch of data was input into the network for 24 samples for network training. The abscissa of the curve represented the training times of the model, and each small cell represented the training times of the model once. The ordinate was the crossentropy loss value of the model prediction result. The loss value was the crossentropy function of the predicted value and the real value during the training process. The vertical axis represented the crossentropy loss value of the model, which reflected the size of the error in the learning process and was used for backpropagation, updating the model parameter values and making the model more accurate. The model was more suitable for the distinction between benign and malignant breast tumors, with an accuracy rate of 99%, and can be continuously extended to practical applications to help clinicians diagnose breast tumors.  Figure 4 is a diagram of one of the cases.

### 3.3. Examination Results

**Figure 5:**
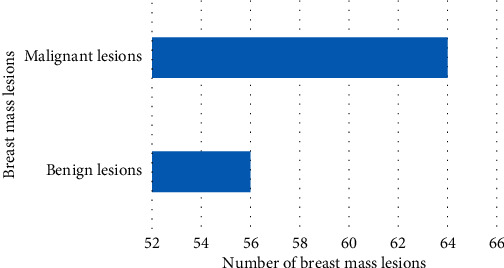
Lesions of breast tumor.

Among 120 cases of breast masses, 47 cases were <0.9 cm lesions and 73 cases were >0.9 cm lesions. In Figure 5, there were 56 cases of benign lesions (41 cases of fibroadenoma, 6 cases of inflammatory lesions, and 9 cases of adenopathy) and 64 cases of malignant lesions (52 cases of invasive ductal carcinoma and 12 cases of intraductal carcinoma).

**Figure 6:**
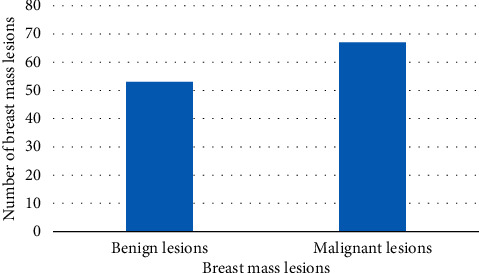
TUI diagnosis of breast tumor lesions.

As shown in Figure 6, 53 cases of benign diseases and 67 cases of malignant diseases were diagnosed by TUI, including 2 cases of fibroadenoma over-diagnosed, 1 case of intraductal papilloma, and 1 case of inflammatory disease. The sensitivity, specificity, accuracy, positive predictive value, and negative predictive value of TUI in the diagnosis of breast cancer were 90.4%, 75.6%, 81.4%, 84.7%, and 80.6%, respectively.

**Figure 7:**
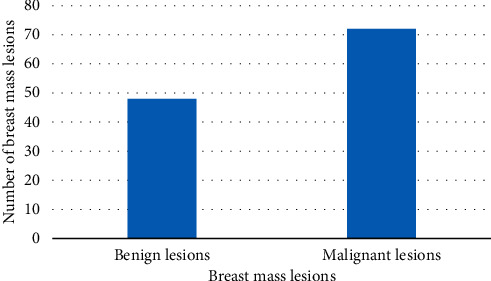
Ultrasound diagnosis of breast tumor lesions.

As shown in Figure 7, ultrasound diagnosis results showed 48 cases of benign diseases and 72 cases of malignancy, including 2 cases of fibroadenoma over-diagnosis, 2 cases of intraductal papilloma, and 3 cases of inflammatory disease. The sensitivity, specificity, accuracy, positive predictive value, and negative predictive value of ultrasound in the diagnosis of breast cancer were 81.7%, 64.9%, 70.5%, 75.9%, and 80.6%, respectively.

**Figure 8:**
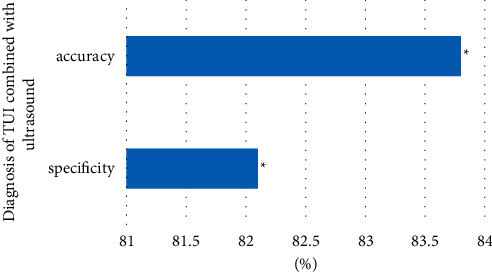
Diagnosis of TUI combined with ultrasound. *Note.* “∗” in the figure indicated that the comparison between the two groups was statistically significant, *P* < 0.05.

In addition, for the bi-RadS4 ultrasound classification (suspected breast malignant lesions), ultrasound was combined with TUI. As shown in Figure 8, the diagnostic specificity increased to 82.1% and the accuracy rate increased to 83.8%. Compared with pure ultrasonography, the sensitivity of combined diagnosis of TUI and ultrasound was higher, and the difference was statistically significant (*P* < 0.05). Comparison on the accuracy of DOT, combined diagnosis showed the difference was not statistically significant (*P* > 0.05).

## 4. Discussion

In the 1970s and 1980s, the application of visible light and near-infrared light in the spectrum attracted people's attention [10]. With the continuous progress of research, researchers believe that future studies of mammary optical technology should include many clinical research samples to obtain the overall sensitivity and specificity of mammary optical imaging [11]. Ultrasonic light scattering breast imaging system is a multimode breast cancer screening, diagnosis, and therapeutic effect detection system based on photon scattering tomography [12]. It integrates ultrasound technology and optical technology into the breast detection device, realizing the organic combination of structural imaging and functional imaging. It can not only determine the location, shape, and internal echo of the mass but also analyze the mass from the functional level without the help of any external contrast agent [13]. TUI is an optical imaging technology that uses near-infrared light to detect the absorption and scattering characteristics of biological tissues to obtain information such as physiological state, biological activity, and molecular function [14]. The important feature of TUI technology is that it can measure and monitor local blood parameters in a noninvasive way [15]. Because TUI can distinguish between scattering and absorption effects in tissues, it makes the quantitative measurement of local blood parameters more accurate. In addition, TUI can perform tomography of local blood parameters [16]. TUI technology was used to diagnose breast tumor. The results showed that the light scattering imaging results were compared with the pathological results, and the diagnosis coincidence rate was 81.4%, which was consistent with the literature report [17].

At present, deep learning technology has been widely used in various fields, such as military, medicine, and geography [18, 19]. Due to the continuous development of imaging technology, a large amount of imaging data has been obtained clinically, but how to mine useful information from the massive imaging data and apply it to the diagnosis of clinical diseases is one of the goals that need to be achieved at present. The emergence of deep learning has effectively reduced the rate of misdiagnosis and missed diagnosis of diseases and reduced the workload of doctors, realizing intelligent diagnosis of diseases [20]. DNN algorithm was used to process TUI images of breast cancer patients, and the results showed that the technique can effectively recognize the lesion areas in the images. The research of Liu et al. [21] and Yuan et al. [22] also showed that the application of DNN algorithm in medical image processing had good effects.

In this study, the specificity of the combined diagnosis of breast malignant lesions by the TUI system and US was 82.1%, and the accuracy rate was increased to 83.8%. Compared with a single examination, the sensitivity of combined diagnosis of TUI and ultrasound was higher than that of ultrasound alone, which was statistically significant. However, compared with light scattering imaging, the sensitivity was not statistically significant, which was related to the high sensitivity of tomography [23]. Breast cancer is mainly invasive ductal carcinoma, and liquefaction necrosis rarely occurs. Therefore, the specificity and accuracy of the diagnosis of TUI combined with US is significantly higher than that of simple ultrasound examination [24].

## 5. Conclusion

In conclusion, TUI combined with ultrasound had a significant effect on benign and malignant diagnosis of breast cancer and can significantly improve the specificity and accuracy of diagnosis. It also reflected that deep learning technology had a good auxiliary role in the examination of diseases and was worth the promotion of clinical application. However, there are also many shortcomings in this work, such as the small sample size of the study, and the clinical trial should not be conducted in a single region or a small area of multicenter and large-sample hospitals. As a promising research direction, deep learning has been widely used in people's life, including the medical industry, and its role in promoting the development of medical technology cannot be underestimated.

## Data Availability

The data used to support the findings of this study are available from the corresponding author upon request.
